# Cell adhesion molecule CD44 is dispensable for reactive astrocyte activation during prion disease

**DOI:** 10.1038/s41598-024-63464-3

**Published:** 2024-06-14

**Authors:** Barry M. Bradford, Lauryn Walmsley-Rowe, Joe Reynolds, Nicholas Verity, Neil A. Mabbott

**Affiliations:** 1 The Roslin Institute and R(D)SVS, University of Edinburgh, Easter Bush Campus, Midlothian, EH25 9RG UK; 2 Maurice Wohl Basic and Clinical Neuroscience Institute, King’s College London, Denmark Hill, London, SE5 9NU UK

**Keywords:** Prion disease, Astrocyte, Microglia, Cell adhesion molecule CD44, Neurodegeneration, Prion diseases, Astrocyte

## Abstract

Prion diseases are fatal, infectious, neurodegenerative disorders resulting from accumulation of misfolded cellular prion protein in the brain. Early pathological changes during CNS prion disease also include reactive astrocyte activation with increased CD44 expression, microgliosis, as well as loss of dendritic spines and synapses. CD44 is a multifunctional cell surface adhesion and signalling molecule which is considered to play roles in astrocyte morphology and the maintenance of dendritic spine integrity and synaptic plasticity. However, the role of CD44 in prion disease was unknown. Here we used mice deficient in CD44 to determine the role of CD44 during prion disease. We show that CD44-deficient mice displayed no difference in their response to CNS prion infection when compared to wild type mice. Furthermore, the reactive astrocyte activation and microgliosis that accompanies CNS prion infection was unimpaired in the absence of CD44. Together, our data show that although CD44 expression is upregulated in reactive astrocytes during CNS prion disease, it is dispensable for astrocyte and microglial activation and the development of prion neuropathogenesis.

## Introduction

Prion diseases, or transmissible spongiform encephalopathies, are prototypic protein-misfolding neurodegenerative diseases. Prion diseases occur across a wide array of mammalian species including humans and display zoonotic potential. Infection can spread via a variety of routes although many natural transmissions occur following oral ingestion of prion contaminated food. The infectious agent in prion diseases is a misfolded conformer of the host prion protein which gains limited resistance to protease digestion^1^. Following uptake from the gut, prions initially replicate within the Peyer’s patches of the host’s immune system prior to infection of the nervous system, a process termed neuroinvasion. Following neuroinvasion the prions spread to and replicate within the central nervous system (CNS), activating glial cell responses and causing synaptic and neuronal loss^2^. The result is a progressive neurodegeneration which is invariably fatal as there are currently no effective treatments.

Astrocytes are the resident support cells of the CNS and play roles in learning and memory formation via regulation of synaptic plasticity^3^. Reactive astrocytosis is a common early pathological feature of prion diseases^4^, other protein-misfolding disorders and generally any insult or infection within the CNS^5^. The timing of astrocyte reactivity is predictive of the survival time during prion disease^6^. The factors regulating astrocyte reactivity during prion disease are currently unknown. During prion disease in the absence of microglia however, astrocyte reactivity is accelerated despite unchanged kinetics of prion accumulation^7^. This enhanced astrocyte reactivity includes increased phagocytic uptake of synapses (similar to synaptic pruning observed in microglia) and upregulated unfolded protein responses^7^. These observations are in accordance with previous studies suggesting that the microglia play a neuroprotective role during prion disease^8,9^ in part by restricting or modulating reactive astrocyte activation via as yet undetermined mechanisms or signalling molecules.

Global upregulation of the intermediate filament glial fibrillary acidic protein (GFAP) is observed within the CNS astrocyte population during prion disease. Investigation of the expression of CD44, another pan-activation marker of astrocytes^10^, revealed profound heterogeneity and distinct neuroanatomical targeting within reactive astrocytes during CNS prion disease^4^. Astrocytes have previously been shown to replicate prions independently^11–14^. The reactive astrocytes expressing high levels of CD44 were also present within sites of prion accumulation, suggesting they are potential reservoirs of prion replication within the CNS^4^ and novel targets for therapeutic intervention during CNS prion disease.

Studies suggest that CD44 is a multi-functional cell adhesion and signalling molecule. CD44 has been shown to interact with several ligands including hyaluronic acid, osteopontin, collagens and matrix metalloproteases^15^ and CD44’s specificity for these regulate its functions^16^. CD44 has been implicated in numerous cellular functions including acting as a phagocytic receptor^17,18^ and regulator of Wnt signalling^19^. In astrocytes CD44 has also been described as a regulator of cell morphology^20^ and reactivity via interaction with the glycoprotein NMB (GPNMB)^21^. A recent study has suggested that neuroinflammation in response to neurodegenerative disease such as the toxin 1-methyl-4-phenyl-1,2,3,6-tetrahydropyridine (MPTP) model of Parkinson’s disease is repressed in CD44-deficient mice^22^.

The expression of CD44 by both astrocytes and microglia has also been described during amyotrophic lateral sclerosis (ALS) progression in SOD1(G93A) mice^23^, and in the microglia associated with the amyloid plaques in APP/PS1 transgenic mice^24^. In the middle cerebral artery occlusion (MCAO) model of focal brain ischemia, CD44 has been shown to regulate serglycin (SRGN)-mediated activation of microglia^25^. However, nothing was known of the role of CD44 expression in astrocytes and microglia during CNS prion disease. Therefore, in the current study we used CD44-deficient mice to determine the role of CD44 in astrocyte and microglial responses and the development of the neuropathology during CNS prion disease. Our data show that CD44 is dispensable for glial activation during prion disease or for prion neuropathogenesis.

## Materials and methods

### Ethics statement

Ethical approvals for the in vivo mouse experiments were obtained from The Roslin Institute’s and University of Edinburgh’s ethics committees. The experiments were designed in accordance with the ARRIVE guidelines, performed under the authority of a UK Home Office Project Licence and in accordance with the guidelines and regulations of the UK Home Office ‘Animals (scientific procedures) Act 1986’. Appropriate care was provided to minimise harm and suffering, and anaesthesia was administered where necessary. Mice were humanely culled at the end of the experiments by cervical dislocation.

### Mice and Prion infection

C57BL/6J wild type (WT) and B6.129(Cg)-Cd44^tm1Hbg^/J mice, Jax stock #:005085^26^ (CD44^−/−^) mice were bred and co-housed under specific pathogen-free conditions. Food and water were provided ad libitum. Groups of 10 mice per genotype of mixed gender were infected at 7–10 weeks old via intracerebral injection with 20 µl of a 1% (weight/volume) brain homogenate prepared from mice terminally infected with ME7 scrapie prions. Groups of 4 mice per genotype were culled at the commencement of overt clinical signs of prion disease at 140 days post infection, the remaining 6 mice per genotype were observed for signs of clinical prion disease as described elsewhere^27^ and culled at a standard clinical end-point. Survival times were calculated as the interval between injection and positive clinical assessment of terminal prion disease. Groups of CD44^−/−^ mice and WT littermate mice (n = 6/group) were used throughout the study and culled alongside terminal prion infected mice to act as age-matched, uninfected controls.

### Gait analysis

Gait analysis was performed weekly using the CatWalkXT (Noldus, Wageningen, the Netherlands) from 8 weeks of age until positive clinical assessment of prion disease. Groups of age and sex-matched uninfected mice of both genotypes were similarly monitored weekly from 8 to 30 weeks of age as controls.

### Neuropathological analysis

Clinical prion disease diagnosis was confirmed by histopathological assessment of vacuolation (spongiform pathology) in the brain. Coronal sections of paraffin-embedded brain tissue were cut at 6 µm thickness, de-paraffinized and stained with hematoxylin & eosin and scored for spongiform vacuolar degeneration as described previously^28^. For the construction of lesion profiles, sections were scored for the presence and severity (scale 0–5) of prion-disease-specific vacuolation in nine grey matter and three white matter areas: G1, dorsal medulla; G2, cerebellar cortex; G3, superior colliculus; G4, hypothalamus; G5, thalamus; G6, hippocampus; G7, septum; G8, retrosplenial and adjacent motor cortex; G9, cingulate and adjacent motor cortex; W1, inferior and middle cerebellar peduncles; W2, decussation of superior cerebellar peduncles; and W3, cerebellar peduncles.

### Immunohistochemistry

Paraffin-embedded brain sections (thickness 6 μm) were deparaffinized and pre-treated by autoclaving in distilled water at 121 °C for 15 min and endogenous peroxidases subsequently quenched by immersion in 4% H_2_0_2_ in methanol for 5 min. For the detection of PrP, Iba1 and Icam-1 sections were autoclaved in target retrieval solution (Dako, Glostrup, Denmark) using an automated 121 °C 15-min cycle. Sections were incubated overnight with primary antibodies; rabbit polyclonal anti Iba1 (Aif1) (Wako, Neuss, Germany), biotin anti mouse CD44 clone IM7 (Biolegend, London, UK), anti-mouse CD54 (ICAM-1) clone YN1/1.7.4 (Biolegend, London, UK), rabbit polyclonal anti GFAP (Dako, Glostrup, Denmark), mouse anti PrP clone BH1^29^ for PrP. Primary antibody binding was detected using biotinylated goat anti species-specific antibodies (Jackson Immunoresearch, Cambridge, UK) where necessary and visualized using the Elite ABC/HRP kit (Vector Laboratories, Peterborough, UK) and diaminobenzidine (DAB) (Merck, Glasgow, UK) between stringent washing steps. Sections were lightly counterstained with hematoxylin and imaged on a Nikon Ni.1 Brightfield Compound upright microscope, 4x/10x/20x/ air lenses, Zeiss 105c colour camera & Zen 2 software for image capture.

### Western blot analysis

Brain homogenates (10% weight/volume) were prepared in NP40 lysis buffer (1% NP40, 0.5% sodium deoxycholate, 150 mM NaCl, 50 mM Tris–HCl [pH 7.5]). For the detection of PrP^Sc^ a sample of homogenate was incubated at 37°C for 1 h with 20 µg/ml proteinase K (PK) and digestion halted by addition of 1 mM phenylmethylsulfonyl fluoride. Samples were denatured at 98°C for 15 min in 1 × SDS sample buffer and separated via electrophoresis through 12% Tris–glycine polyacrylamide NuPAGE™ gels (ThermoFisher Scientific, Winsford, UK) and transferred to polyvinylidene difluoride PVDF membranes by semi-dry electroblotting. Primary antibodies; mouse anti PrP clone BH1^29^ for PrP, and beta actin (C4) (Santa Cruz Biotechnology, Santa Cruz, CA, USA) were detected by horseradish peroxidase-conjugated goat anti-species specific antibody (Jackson Immunoresearch, Cambridge, UK) and visualized via chemiluminescence (BM Chemiluminescent substrate kit, Roche, Welwyn Garden City, UK) as described previously^30^. Western blot images were acquired using a GeneGnome XRQ chemiluminescence imaging system using Genesys V1.6.10 software (Syngene, Cambridge). Optimal exposure time was applied within Genesys software to ensure sample signal was not saturated/overexposed. Images were inverted to display black signal on white background. No other image enhancement, contrast or other adjustments were applied. The complete western blot images for all samples are provided in Supplemental Fig. 1.

**Figure 1:**
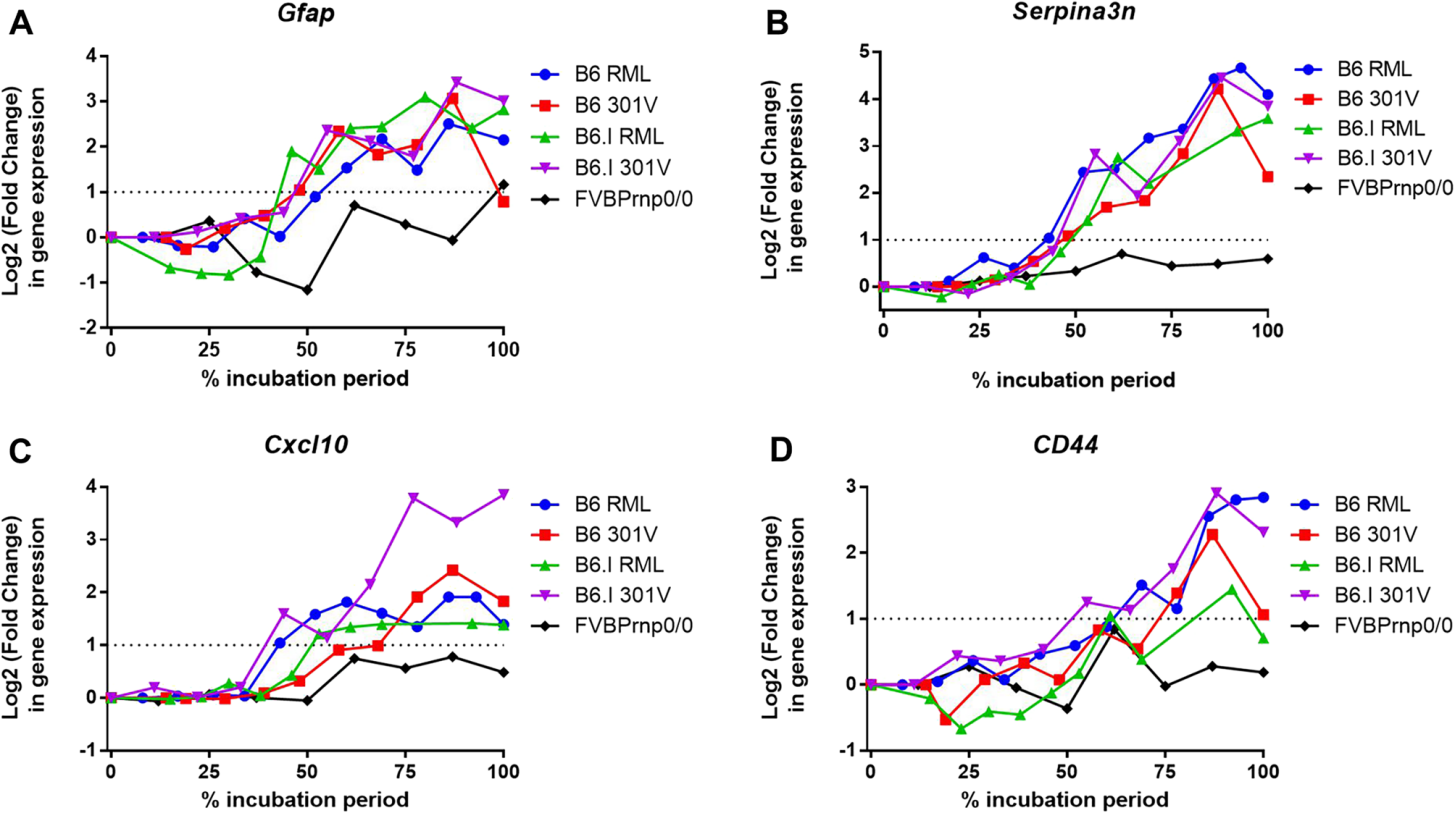
Astrocyte reactivity during prion disease. Uniform upregulation of (**A**) *Gfap*, (**B**) *Serpina3n*, (**C**) *Cxcl10* and (**D**) *Cd44* across varied prion strain/host combinations visualised by plotting log2 fold change in gene expression versus % incubation period. Expression data were derived from a microarray dataset of gene expression in the brains of mice infected with distinct prion isolates^30^.

### Image analyses

Image analysis was performed using ImageJ software (https://imagej.net/ij/) (Schneider et al., 2012) and Imaris software (Oxford Instruments, Abingdon, UK). Astrocyte morphology was assessed using Imaris filament tracing. The magnitude of PrP^d^, GFAP and CD44 immunostaining on DAB stained sections was compared as previously described^4^. Briefly, the optical density (OD) values for immunostaining were calculated using ImageJ software following H-DAB deconvolution. Mean grey OD values were measured from DAB grayscale images (scaled 0–255) and expressed as a % relative intensity by dividing by the maximum value (255). For astrocyte density GFAP-stained images of hippocampus were colour-deconvoluted to isolate DAB staining, subject to intensity threshold and particle analysis to obtain total astrocyte number, density was calculated by measuring the area of the hippocampus and expressing as number of cells/area. Western blot images were subjected to densitometric analyzed by ImageJ. Briefly lanes and bands were identified, threshold levels set and area under the curve measurements taken (pixels). For PrP^C^ and PrP^Sc^ relative expression levels were calculated as a percentage relative to a control normal brain PrP^C^ measurement.

### Gene expression analysis via RT-qPCR

Total RNA was isolated from brain using RNABee (AMSBio, Abingdon, UK) and RNeasy Mini kit (Qiagen, Manchester, UK). RNA was DNase treated (Promega, Southampton, UK) to remove genomic DNA. Reverse transcription of polyA mRNA from 5 µg total DNA-free RNA was performed using Superscript First Strand Synthesis (ThermoFisher Scientific, Winsford, UK) with Oligo-dT primers. Quantitative PCR (qPCR) were performed using SYBR master mix (Rox) (Roche, Welwyn Garden City, UK) on an MX3005pro (Agilent Technologies, Wokingham, UK) using the primer sequences detailed (Table 1). Gene expression relative to naïve wild-type mice was calculated using the ΔΔCT method^31^ using *Rpl19* as a reference gene, a mean of ≥ or ≤ 2.0-fold change and p-value of ≤ 0.05 were considered significant.

**Table 1:** qPCR primers.

Target	Sequence
*Aif1* forward	GGATCAACAAGCAATTCCTCGA
*Aif1* reverse	CTGAGAAAGTCAGAGTAGCTGA
*Cd44* forward	ACCTTGGCCACCACTCCTAA
*Cd44* reverse	GCAGTAGGCTGAAGGGTTGT
*Gfap* forward	AGAAAGGTTGAATCGCTGGA
*Gfap* reverse	CGGCGATAGTCGTTAGCTTC
*Il1b* forward	CTGAACTCAACTGTGAAATGCCA
*Il1b* reverse	AAAGGTTTGGAAGCAGCCCT
*Il6* forward	ACCAGAGGAAATTTTCAATAGGC
*Il6* reverse	TGATGCACTTGCAGAAAACA
*Psmb8* forward	CAGTCCTGAAGAGGCCTACG
*Psmb8* reverse	CACTTTCACCCAACCGTCTT
*Rpl19* forward	GAAGGTCAAAGGGAATGTGTTCA
*Rpl1* reverse	CCTTGTCTGCCTTCAGCTTGT
*Serpina3n* forward	CCTGGAGGATGTCCTTTCAA
*Serpina3n* reverse	TTATCAGGAAAGGCCGATTG
*Srgn* forward	GCAAGGTTATCCTGCTCGGA
*Srgn* reverse	TGGGAGGGCCGATGTTATTG
*Tgfb* forward	CCACCTGCAAGACCATCGAC
*Tgfb* reverse	CTGGCGAGCCTTAGTTTGGAC
*Tnf* forward	TGTGCTCAGAGCTTTCAACAA
*Tnf* reverse	CTTGATGGTGGTGCATGAGA

### Statistical analyses

Statistical analyses were performed using GraphPad Prism (Dotmatics, Boston, MA, USA). Survival curve analysis performed by Log-rank [Mantel-Cox] Test. Image analyses performed by ANOVA with Tukey’s multiple comparison test. qPCR analyses were performed by ANOVA with Sidak’s multiple comparison test results expressed as dot plots of individual animal observations with mean values indicated (bar). CatWalk XT analysis performed using Two-Way ANOVA and expressed as group mean with 95% confidence interval. Values of *P* < 0.05 were accepted as significant.

## Results

### Induction of CD44 expression coincides with the onset of reactive astrocyte activation

Previous studies show that the timing of the onset of the astrocyte reactivity is predictive of the survival time during prion disease^4,6^. To further explore the relationships between the transcriptional activation of the reactive astrocytes and CNS prion disease survival time, we analysed a large publicly available microarray dataset of gene expression in the brains of mice infected with distinct prion isolates^32^. This analysis revealed remarkable uniformity in the upregulation of expression of several reactive-astrocyte activation associated genes as the disease progressed, including *Gfap* (Fig. 1A), *Serpina3n* (Fig. 1B) and *Cxcl10* (Fig. 1C). Despite survival times ranging from 112 to 294 days across these different prion strain/host combinations, each of these reactive astrocyte-associated genes were statistically significantly upregulated from approximately 45% of the way through the survival time period. We have previously described astrocyte heterogeneity via CD44 expression and how this describes the precise neuropathological targeting of each prion strain/host combination^4^. Despite this heterogeneity, this analysis also showed that *Cd44* expression was significantly upregulated in most of the prion/host combinations studied from approximately 50% of the way through prion disease survival time (Fig. 1D).

### The onset of CNS prion disease is unaltered in the absence of CD44

**Figure 2:**
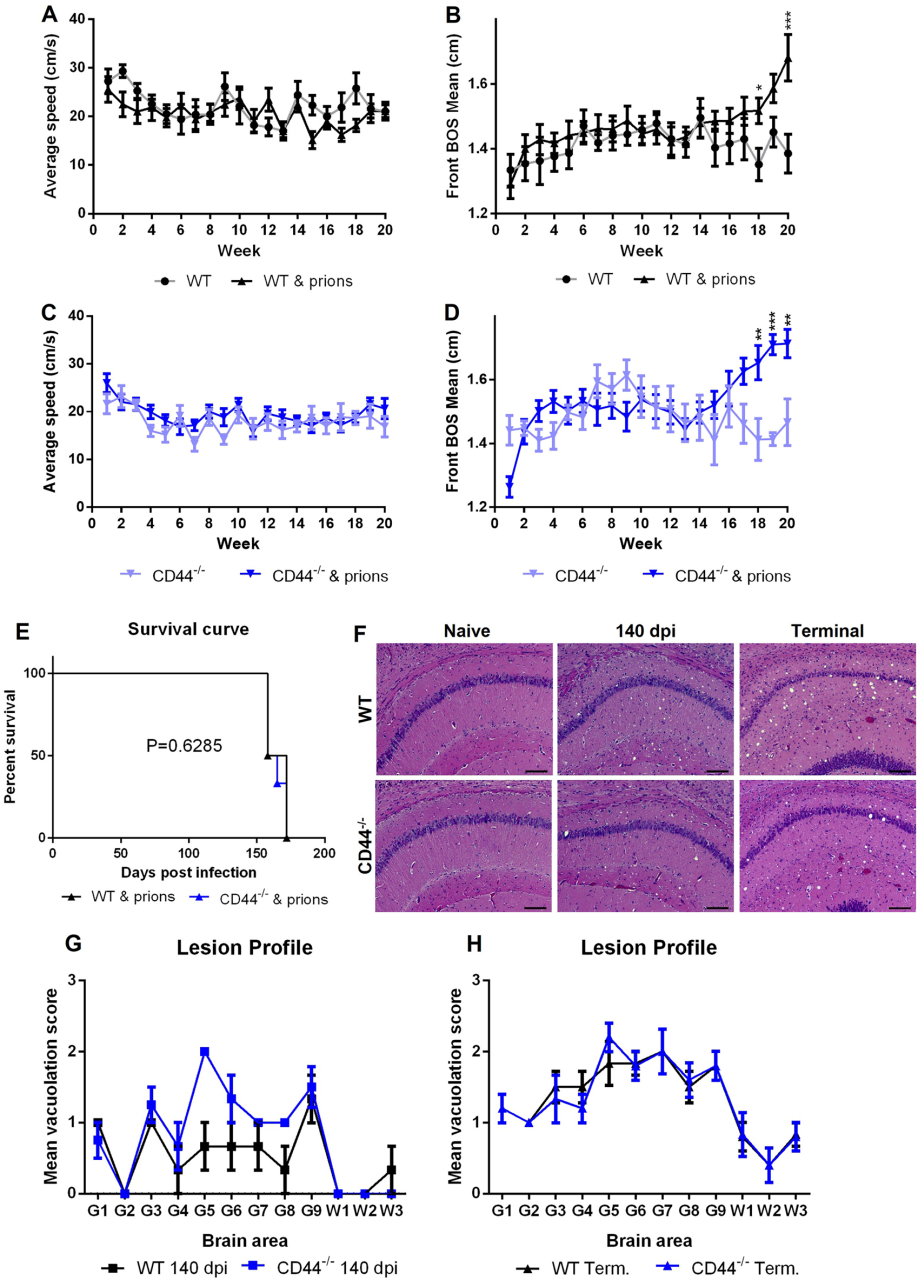
Response to CNS prion disease is unaltered in CD44-deficient mice. (**A**) CatWalk gait analysis weekly assessment of average speed and (**B**) front base of stance (BOS) of naïve vs prion infected WT mice. (**C**) CatWalk gait analysis average speed and (**D**) front BOS of naïve vs prion infected CD44^−/−^ mice. Points represent group mean and error bars 95% confidence interval. Two-Way ANOVA with Tukey’s multiple comparison test. *P < 0.05; **P < 0.005; ***P < 0.001. N = 6–10 mice/group. (**E**) Survival curve analysis of WT and CD44^−/−^ mice following intracerebral prion infection, Log-rank Mantel Cox Test. N = 6 mice/group. (**F**) Representative images from hematoxylin and eosin-stained hippocampus CA1 of naïve, 140 dpi or terminal prion infected WT and CD44^−/−^ mice. Scale bar = 100 µm. (**G**) Lesion profile analysis of 140 dpi prion-infected brains. Points represent the mean vacuolation score, error bars =  ± SEM. Two-way ANOVA, Sidak's multiple comparisons test, N = 4 mice/group. (**H**) Lesion profile analysis of terminal prion-infected brains. Points represent the mean vacuolation score, error bars =  ± SEM. Two-way ANOVA, Sidak's multiple comparisons test, N = 6 mice/group.

To determine the role of CD44 in prion disease, C57Bl/6J wild type (WT) and CD44-deficient (CD44^−/−^) mice^26^ were injected intracerebrally with ME7 mouse-adapted scrapie prions. Following injection, prion infected and naïve uninfected mice were assessed weekly via CatWalk automated gait analysis and scored for clinical signs of prion disease. The average speed of WT mice crossing the CatWalk was unimpaired by prion infection (Fig. 2A).  However gait disturbance displayed by divergence of front base of stance commenced around 14 weeks post prion infection in WT mice and was statistically significantly different from uninfected mice after 18 weeks post infection (Fig. 2B). Analysis of prion infected CD44^−/−^ mice also revealed no effect on average speed (Fig. 2C) and similar disturbance to front base of stance becoming significant 18 weeks post infection (Fig. 2D). These data revealed that CD44 deficiency had no impact on the timing of the prion induced gait disturbance. No differences were observed in the clinical presentation of prion disease and prion disease survival time between WT (165 ± 8 days; mean ± SD) and CD44^−/−^ (164 ± 7 days) mice (Fig. 2E).

To determine the impact of CD44-deficiency on the development of the neuropathology, we assessed the magnitude of the prion-disease specific vacuolation in hematoxylin and eosin-stained brain sections (Fig. 2F) across 12 standard areas. This analysis suggested that the targeting and severity of the prion-induced vacuolation was similar in the brains of the terminally-affected WT and CD44^−/−^ mice at both 140 days post infection (dpi) (Fig. 2G) and terminal timepoints (Fig. 2H).

### Unaltered prion accumulation in the brain in the absence of CD44.

**Figure 3:**
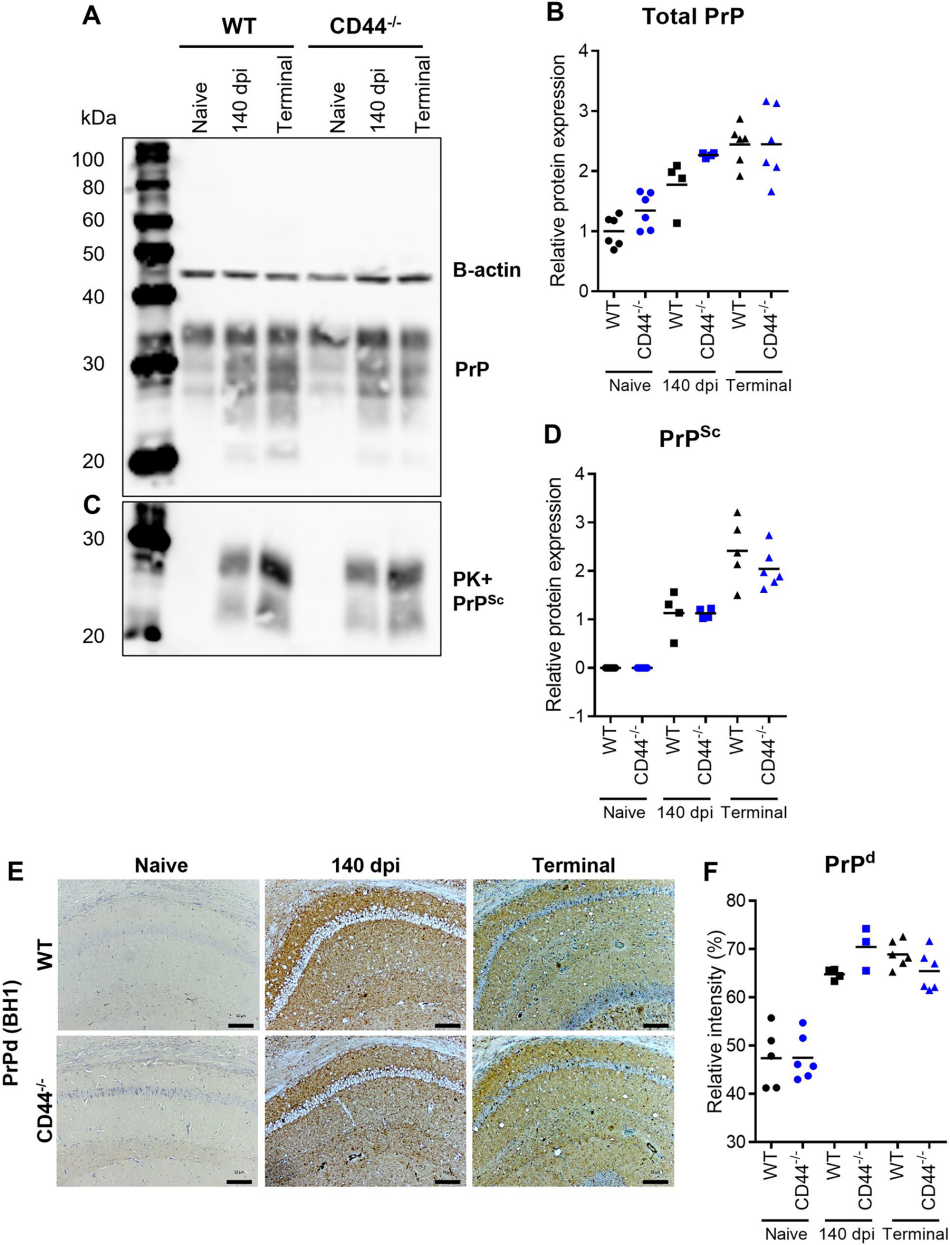
Prion protein expression and prion accumulation and distribution are unaltered in the brains of CD44-deficient mice. (**A**) Western blot analysis of representative WT and CD44^−/−^ mice mouse brain from naïve, 140 dpi and terminal prion infection, probed with anti-PrP antibody clone BH1, relative protein sizes indicated in kilodaltons (kDa). (**B**) Quantitation of relative total PrP levels normalised to beta-actin. Points represent individual mice. WT (Black) and CD44^−/−^ (Blue) naïve (circle) 140 dpi (square) and terminal (triangle) mice/groups as indicated; bar represents group mean. (**C**) Western blot analysis of representative WT and CD44^−/−^ mice mouse brain from naïve, 140 dpi and terminal prion infection following limited proteinase K (PK) digestion. (D) Quantitation of relative proteinase K resistant PrP^Sc^ levels. (**E**) Representative images from PrP immunostained hippocampus CA1 of naïve, 140 dpi or terminal prion infected WT and CD44^−/−^ mice. Scale bar = 100 µm. (**F**) Quantitation of relative intensity of PrP staining. ANOVA with Tukey’s multiple comparison test.

During prion disease astrocytes accumulate prions and may be sites of prion replication. We have previously positively correlated high CD44 expression with prion accumulation in astrocytes in the brains of mice infected with distinct mouse adapted prion strains^4^. We therefore assessed the impact of CD44-deficiency on prion accumulation in the brain. Infectious prions are considered to comprise of misfolded forms of the cellular prion protein PrP^C^, and the expression of PrP^C^ in the brain can temporally control prion disease survival time^33^. Since CD44 and PrP^C^ have been shown to functionally interact in multidrug resistant breast cancer cells^34^, we first assessed the impact of CD44-deficiency on the expression level of PrP^C^ in the brain. Western blot analysis suggested no differences in the expression of PrP^C^ in the brains of WT and CD44^−/−^ mice (Fig. 3A,B). The accumulations of abnormally folded, prion disease-specific, relatively proteinase K (PK)-resistant PrP (termed PrP^Sc^) were also similar in the brains of WT and CD44^−/−^ mice following prion infection (Fig. 3C,D). Immunostaining to detect these abnormal, prion disease specific accumulations of PrP in the brain (termed PrP^d^), similarly revealed no differences in their abundance or distribution in the brains of WT and CD44^−/−^ mice at either 140 dpi or terminal timepoint (Fig. 3E,F).

### Impact of CD44-deficiency on glial cell responses

**Figure 4:**
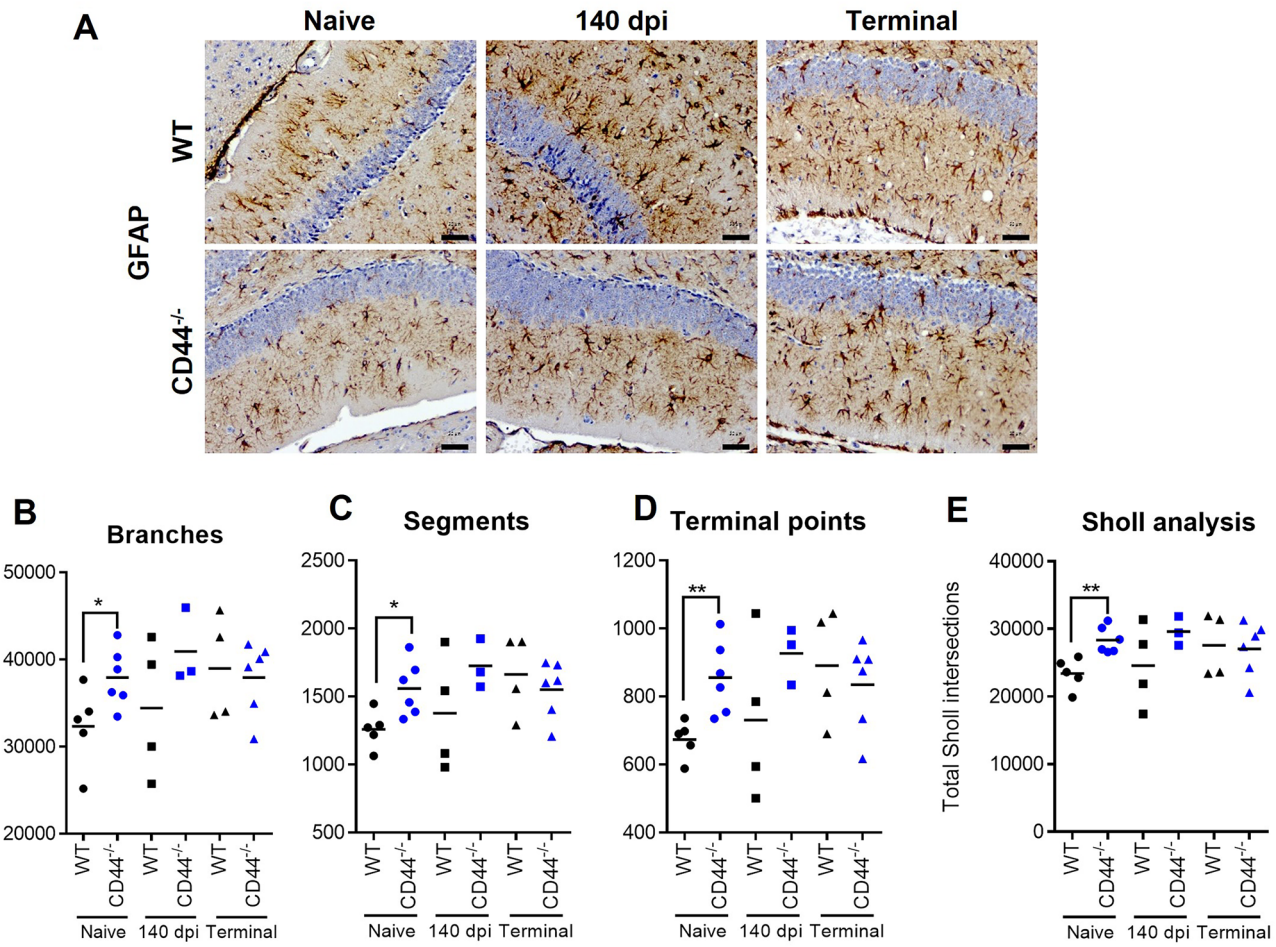
Astrocyte morphological assessment in CD44-deficient mice. (**A**) Representative images from GFAP immunostained Dentate Gyrus molecular layer of naïve, 140 dpi or terminal prion infected WT and CD44^−/−^ mice. Scale bar = 20 µm. Total number of (**B**) branches, (**C**) segments, (**D**) terminal points and (**E**) Sholl intersections as determined by Imaris filament tracing. Points represent individual mice, bar = group mean. ANOVA with Sidak’s multiple comparison test. *P < 0.05; **P < 0.005.

Next, the impact of CD44 deficiency on microglia and astrocyte activation in the brains of prion infected mice was assessed. To address the potential role of CD44 in astrocyte morphology we used Imaris software to perform filament tracing analysis on glial fibrillary acidic protein (GFAP) immunostained sections of the molecular layer of the dentate gyrus, an area minimally affected by extra-cytoskeletal accumulation of GFAP during prion infection (Fig. 4A). In WT mice, prion infection results in a subtle increase in astrocyte complexity. CD44^−/−^ mice displayed high GFAP morphological complexity in the naïve state including greater branching, segments, terminal point and Sholl intersections when compared to naïve WT mice which remained unchanged during prion infection (Fig. 4B–E).

**Figure 5:**
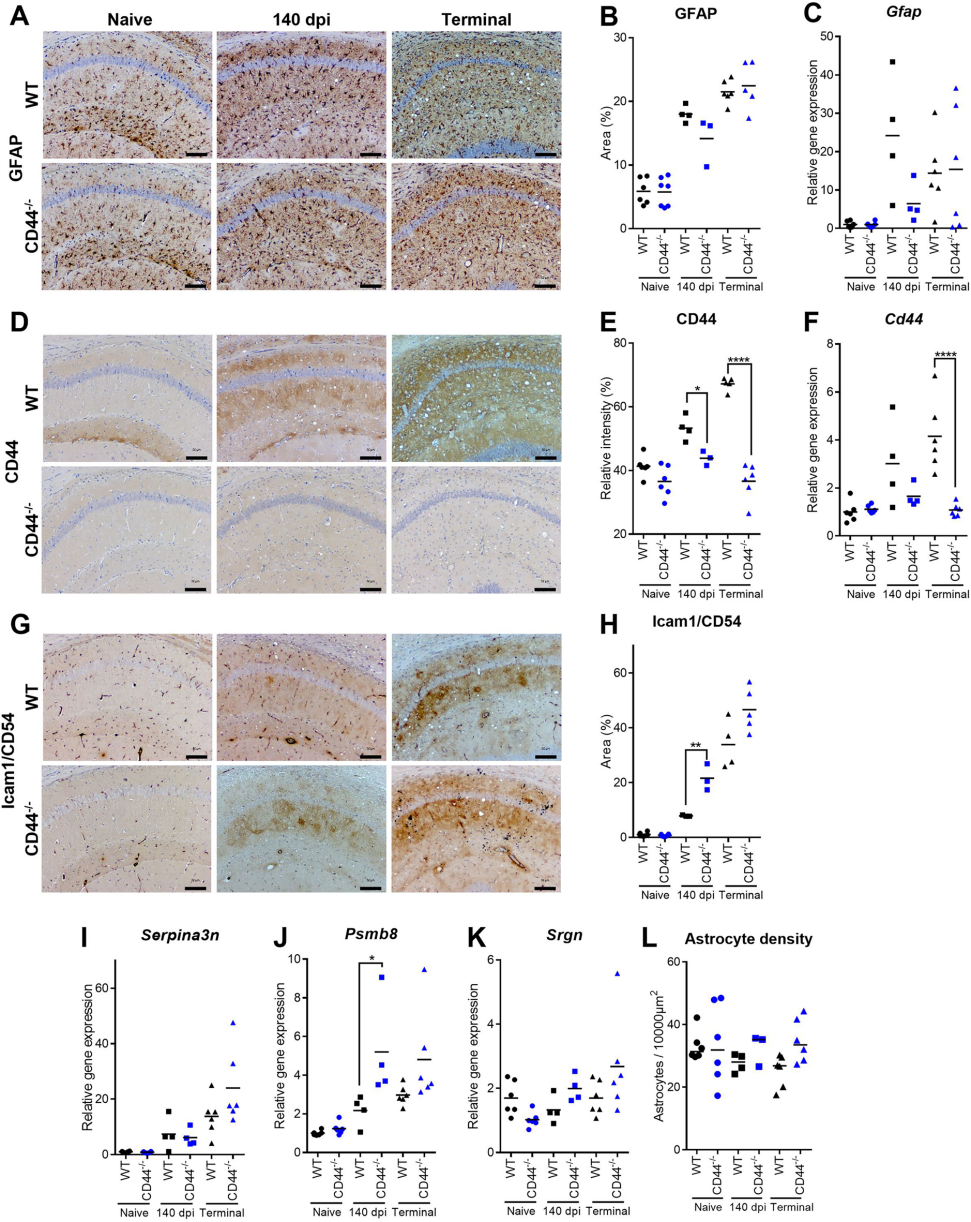
Astrocyte responses to CNS prion disease. (**A**) Representative images from GFAP immunostained hippocampus CA1 of naïve, 140 dpi or terminal prion infected WT and CD44^−/−^ mice. Scale bar = 100 µm. (**B**) Quantitation of % area coverage of GFAP staining. (**C**) RT-qPCR analysis of *Gfap* gene expression. (**D**) Representative images from CD44 immunostained hippocampus CA1 of naïve, 140 dpi or terminal prion infected WT and CD44^−/−^ mice. Scale bar = 100 µm. (**B**) Quantitation of relative intensity of CD44 staining. (C) RT-qPCR analysis of *Cd44* gene expression. (**G**) Representative images from Icam1 immunostained hippocampus CA1 of naïve, 140 dpi or terminal prion infected WT and CD44^−/−^ mice. Scale bar = 100 µm. (**H**) Quantitation of % area coverage of Icam1 staining. RT-qPCR analysis of (**I**) *Serpina3n*, (**J**) *Psmb8* and (**K**) *Srgn* gene expression. (**L**) Quantitation of astrocyte density within the hippocampus calculated from the number of GFAP+ cells per area. Points represent individual mice, bar = group mean. ANOVA with Sidak’s multiple comparison test. *P < 0.05; **P < 0.005; ****P < 0.0001.

Further analysis of prion-targeted areas such as the CA1 of the hippocampus suggested there was no difference in the magnitude of GFAP^+^ immunostaining in brains of prion infected WT and CD44^−/−^ mice (Fig. 5A,B). The expression of *Gfap* mRNA was likewise upregulated in the brains WT mice and CD44^−/−^ mice in response to prion infection (Fig. 5C). Immunostaining for CD44 revealed progressive accumulation in prion infected WT mice. In contrast in CD44^−/−^ mice following prion infection CD44 was undetectable as expected (Fig. 5D,E). The expression of *Cd44* mRNA was similarly significantly upregulated fourfold in response to prion infection in the brains of terminal WT mice, but not in the brains of prion infected CD44^−/−^ mice (Fig. 5F). We have previously identified heterogenic responses of astrocytes to prion infection using upregulation of CD44 expression^4^. Intercellular adhesion molecule 1 (Icam1/CD54) upregulation has also been shown in reactive astrocytes in response to tumor necrosis factor alpha (TNFα), interleukin 1 beta (Il1β) and interferon gamma (IFNγ) signalling^35,36^. To study astrocyte heterogeneity in the absence of CD44, we performed immunostaining for Icam1 in brains from WT and CD44^−/−^ mice (Fig. 5G). Similar to CD44, Icam1 was undetectable in naïve mice. In WT mice Icam1 accumulation was observed with a similar heterogenic and astrocytic pattern to CD44 in targeted brain areas such as the hippocampus at the terminal stage of prion disease. In CD44^−/−^ mice Icam1 expression was also evident and significantly upregulated at the pre-clinical timepoint compared to WT mice. However, this difference in Icam1^+^ immunostaining between CD44^−/−^ and WT mice was not significant at the terminal stage of prion disease (Fig. 5G,H). These data indicate astrocyte heterogeneity was maintained in the absence of CD44 expression in prion infected CD44^−/−^ mice. Assessment of the expression of other reactive astrocyte-associated genes such as *Serpina3n* (Fig. 5I) *Psmb8* (Fig. 5J) and *Srgn* (Fig. 5K) revealed equivalent upregulation in response to prion infection in WT and CD44^−/−^ mice at both timepoints suggesting the accumulation of Icam1 at 140 dpi was not indicative of greater astrocyte reactivity in general at this timepoint but perhaps a more specific response in the absence of CD44. In order to quantify changes in astrocyte-associated gene expression we assessed the relative abundance of astrocytes within the hippocampus. We observed no statistically significant difference in astrocyte abundancy between WT and CD44^−/−^ mice or during prion disease (Fig. 5L) in accordance with previous studies that suggested no change in astrocyte abundancy during prion disease^37^. This revealed that the changes in *Cd44* mRNA expression and other astrocyte-associated genes described above were not simply due to an equivalent increase in the number of astrocytes in the brain during CNS prion disease.

**Figure 6:**
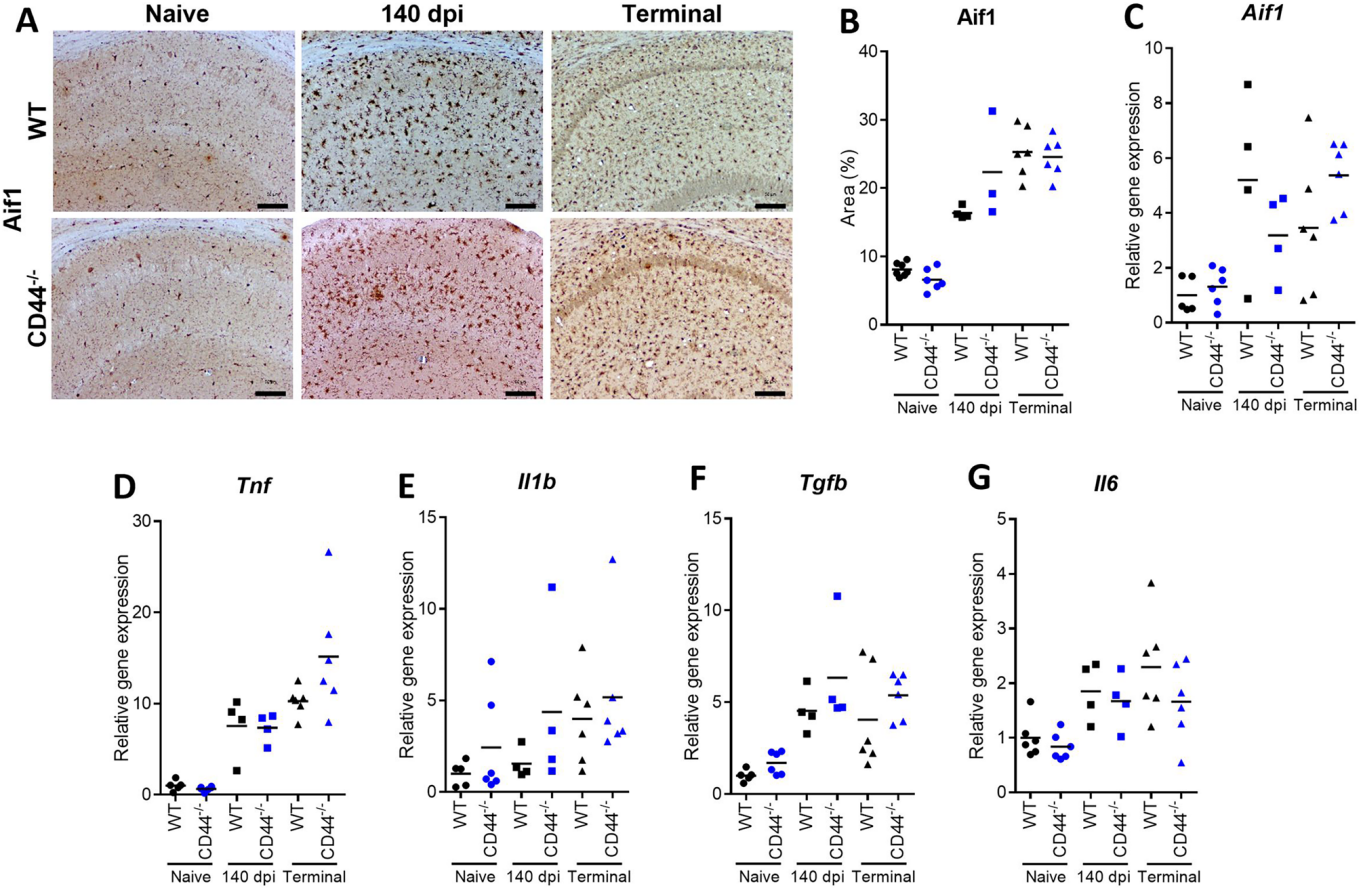
Microglial responses to CNS prion disease. (**A**) Representative images from Aif1 immunostained hippocampus CA1 of naïve, 140 dpi or terminal prion infected WT and CD44^−/−^ mice. Scale bar = 100 µm. (**B**) Quantitation of % area coverage of Aif1 staining. (**C**) RT-qPCR analysis of *Aif1* gene expression. RT-qPCR analysis of (**D**) *Tnf*, (**E**) *Il1b* and (**F**) *Tgfb*, (**G**) *Il6* gene expression. Points represent individual mice, bar = group mean. ANOVA with Sidak’s multiple comparison test.

Immunostaining for allograft inflammatory factor 1 (AIF1) revealed increased proliferation and altered morphology indicative of microglial activation in prion infected WT mice as has been described previously^7^. CD44 has been shown to play a role in stimulating chemotaxis^38^ and to also act as a phagocytic receptor^17,39^. A recent study has revealed the role of CD44 in the SRGN-mediated activation of microglia during MCAO model of focal brain ischemia in mice^25^. We have previously shown no change in prion accumulation in prion infected microglial-deficient Csf1r^∆FIRE^ KO mice^7^ indicating that microglial-mediated phagocytosis and clearance of prions is negligible during prion disease. CD44^−/−^ mice showed similar microglia histopathological responses to prion infection (Fig. 6A,B). Microglial distribution and morphology were each similar in the brains of prion-infected WT and CD44^−/−^ mice, suggesting no impairment of microglial activation or transit towards prion-targeted areas. Gene expression analysis via RT-qPCR also revealed induction of microglial genes in response to prion infection including upregulation of *Aif1* (Fig. 6C). However, no differences were observed in the upregulation of *Tnf*, *Il1b*, *Tgfb* and *Il6* (Fig. 6D–G)*.* These data suggest that the microgliosis that accompanies CNS prion infection was unimpaired in the absence of CD44.

## Discussion

Astrocyte reactivity is a common feature of prion diseases and their role in prion disease is becoming increasingly clear. Astrocytes critically regulate the timing of prion pathogenesis and specifically targeting astrocyte responses such as inhibiting their unfolded protein response (UPR) can be almost as effective as global inhibition^40^ highlighting their importance as potential therapeutic targets. We and others have previously shown that the cell adhesion molecule CD44 is expressed highly in reactive astrocytes in the prion disease-affected brain^4,32,41–43^. Since data on the transcriptional analysis of brains from human Parkinson’s disease patients have also shown correlation between *Cd44* overexpression and astrocyte activation^44^, this raised the hypothesis that CD44 may play a role in the development of the neuropathology in certain CNS disorders including prion diseases. However, using CD44^−/−^ mice we show here that prion neuropathogenesis and glial cell activation was unaltered in the complete absence of CD44. The development of CNS prion disease and, the magnitude of the PrP^Sc^ accumulation, prion induced vacuolation, microgliosis and reactive astrocytosis in the brains of prion-infected CD44^−/−^ mice were similar to those observed in prion infected WT mice. Together, our data clearly show that CD44 expression in astrocytes during CNS prion disease is dispensable for the development of the reactive astrocytosis and neuropathology.

Whether the expression of other genes can compensate for the deficiency in *Cd44* in the brain during prion infection or other CNS disorders remains to be determined. Perhaps as identified in other experiments using CD44^−/−^ mice, the expression of Icam1/CD54 and other receptors may compensate for the absence of CD44. For example, c-Met has been shown to recruit Icam1 as a co-receptor in CD44^−/−^ mice^45^. Expression of the receptor for hyaluronan-mediated motility (RHAMM) has also been shown to compensate for the increased accumulation of hyaluronic acid in CD44^−/−^ mice^46^. Our observation that Icam1 expression is upregulated to a greater degree at 140 dpi in CD44^−/−^ mice compared to WT mice during prion disease would support the hypothesis that deficiency in CD44 may be compensated for by Icam1 activity. Combined with our observation that astrocyte morphological complexity is maintained in naïve CD44^−/−^ mice it is plausible that developmental and functional redundancies may be compensating for the absence of CD44 in these mice.

In conclusion, further studies are required to determine the factors that mediate the activation of glial responses during CNS prion disease. Treatments that target the neurotoxic activation of reactive astrocytes have been shown to impede the development of CNS prion disease^40^. We have also shown how astrocyte reactivity is regulated by microglia during prion disease via as yet undefined mechanisms^7^. Thus, a thorough understanding of the molecular factors that can modulate astrocyte and microglial activity during CNS prion disease could help identify novel treatments to delay or prevent the progression of prion disease and other similar neurodegenerative disorders.

### Supplementary Information

Supplementary Information.

## Data Availability

All data generated or analysed during this study are included in this published article (and its Supplementary Information files).
