# Supplementary material for: Cell adhesion molecule CD44 is dispensable for reactive astrocyte activation during prion disease
Source: Sci Rep. 2024 Jun 14;14:13749. doi: 10.1038/s41598-024-63464-3 (PMC11178777; doi:10.1038/s41598-024-63464-3)
Supplement: Supplementary file 1 — Supplementary Information. [file 41598_2024_63464_MOESM1_ESM.pdf]

Cell adhesion molecule CD44 is dispensable for reactive astrocyte activation during prion disease

Barry M. Bradford, Lauryn Walmsley-Rowe, Joe Reynolds, Nicholas Verity & Neil A. Mabbott

Supplemental Figure 1. Un-cropped Western blot images for proteinase K untreated and treated brain homogenates from all experimental samples coded and randomised, probed with anti-PrP (BH1) and anti-β actin (C4). Protein expression levels analyzed and presented in Figures 3C & 3D Uncropped composite western blot as displayed in Figure 3A & 3C, red boxes indicate cropped regions used. Western blot images were acquired using a GeneGnome XRQ chemiluminescence imaging system using Genesys V1.6.10 software (Syngene, Cambridge). Optimal exposure time was applied within Genesys software to ensure sample signal was not saturated/overexposed. Images were inverted to display black signal on white background. No other image enhancement, contrast or other adjustments were applied.

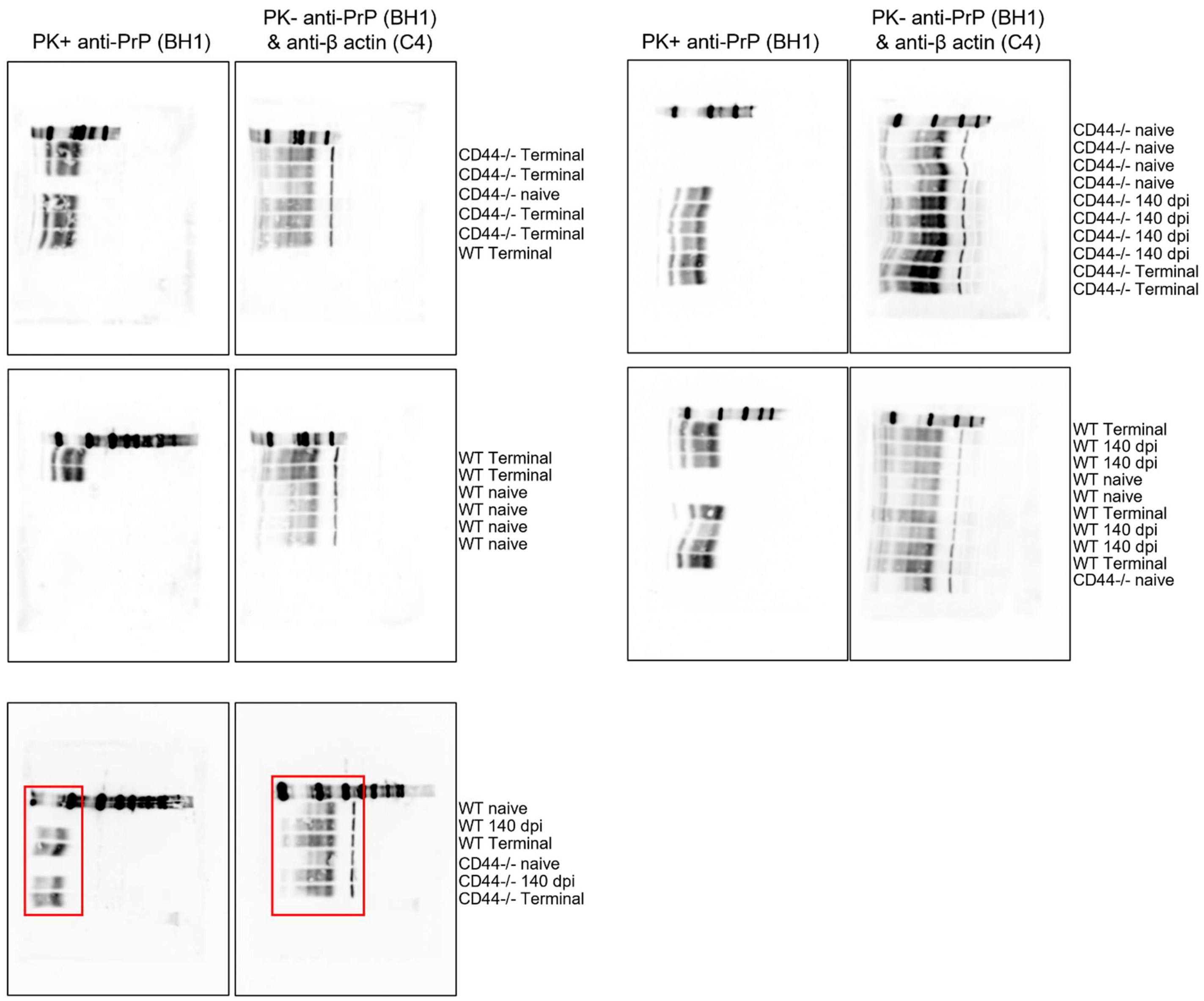
PK+ anti-PrP (BH1)
PK- anti-PrP (BH1) & anti-β actin (C4)
CD44-/- Terminal
CD44-/- Terminal
CD44-/- naive
CD44-/- Terminal
CD44-/- Terminal
WT Terminal
WT Terminal
WT Terminal
WT naive
WT naive
WT naive
WT naive
WT naive
WT 140 dpi
WT Terminal
CD44-/- naive
CD44-/- 140 dpi
CD44-/- Terminal
PK+ anti-PrP (BH1)
PK- anti-PrP (BH1) & anti-β actin (C4)
CD44-/- naive
CD44-/- naive
CD44-/- naive
CD44-/- naive
CD44-/- 140 dpi
CD44-/- 140 dpi
CD44-/- 140 dpi
CD44-/- 140 dpi
CD44-/- Terminal
CD44-/- Terminal
WT Terminal
WT 140 dpi
WT 140 dpi
WT naive
WT naive
WT Terminal
WT 140 dpi
WT 140 dpi
WT Terminal
CD44-/- naive
